# Attitudes towards COVID-19 Vaccination among Hospital Staff—Understanding What Matters to Hesitant People

**DOI:** 10.3390/vaccines9050469

**Published:** 2021-05-06

**Authors:** Anne Spinewine, Catherine Pétein, Perrine Evrard, Christelle Vastrade, Christine Laurent, Bénédicte Delaere, Séverine Henrard

**Affiliations:** 1 Clinical Pharmacy Research Group, Louvain Drug Research Institute, Université Catholique de Louvain, 1200 Brussels, Belgium; catherine.petein@uclouvain.be (C.P.); perrine.evrard@uclouvain.be (P.E.); severine.henrard@uclouvain.be (S.H.); 2 Pharmacy Department, Université Catholique de Louvain, CHU UCL Namur, 5530 Yvoir, Belgium; 3 Nursing Department and Infection Control Unit, Université Catholique de Louvain, CHU UCL Namur, 5530 Yvoir, Belgium; christelle.vastrade@uclouvain.be; 4 Infection Control Unit, Université Catholique de Louvain, CHU UCL Namur, 5530 Yvoir, Belgium; christine.laurent@uclouvain.be; 5 Infectious Disease Department, Université Catholique de Louvain, CHU UCL Namur, 5530 Yvoir, Belgium; benedicte.delaere@uclouvain.be; 6 Institute of Health and Society (IRSS), Université Catholique de Louvain, 1200 Brussels, Belgium

**Keywords:** COVID-19, vaccination, hospital staff, attitudes, vaccine hesitancy, survey

## Abstract

Hospital staff are a priority target group in the European COVID-19 vaccination strategy. Measuring the extent of COVID-19 vaccination hesitancy and understanding the reasons behind it are essential to be able to tailor effective communication campaigns. Using the Health Belief Model (HBM) as a theoretical framework, a survey was conducted among staff members of a Belgian three-site hospital center between 6 and 20 January 2021. Multivariable logistic ordinal regression was performed to assess determinants of the attitude towards COVID-19 vaccination. Reasons for and against COVID-19 vaccination and the need for information were explored among hesitant staff members. Among the respondents (*N* = 1132), 58% and 4.9% said that they would certainly and certainly not get vaccinated, respectively; 37.1% were hesitant, with different degrees of certainty. A positive attitude towards COVID-19 vaccination was associated with being older, being a physician, being vaccinated against seasonal flu, and with several HBM factors (including perceived benefits and cues to actions). Among hesitant staff, concerns about potential side effects and the impression that the vaccine was developed too quickly were the main reasons against COVID-19 vaccination. The key factors in the decision process were data on vaccine efficacy and safety, and knowing that vaccination went well in others. These data are helpful to further tailor the communication campaign and ensure sufficient vaccination coverage among hospital staff.

## 1. Introduction

As of December 2020, more than 18 million individuals had been infected with COVID-19 in Europe and around 412,000 had died [1], of which 17,000 were in Belgium [2]. The pandemic had had a serious impact on hospital burden and on the working conditions and mental health of healthcare professionals (HCPs) [3,4,5]. Yet vaccination was under way, with the first COVID-19 vaccines being evaluated and approved by the European Medicines Agency and the European Commission, and national and international vaccination strategies being developed.

As part of the European vaccination strategy, healthcare workers were identified as one of the priority groups for vaccination, with the goal of vaccinating 80% of them by March 2021 [6]. Accordingly, the Belgian health authorities decided that hospital staff would be included in the first vaccination phase, with the aim of starting to vaccinate HCPs in hospitals around the end of January, and non-HCP hospital staff in February [7].

Several recent studies have shown that a significant proportion of HCPs are hesitant about COVID-19 vaccination. The study conducted by Verger et al. among HCPs in Quebec, France, and Belgium in October and November 2020 showed that 43.8% of the HCPs surveyed would “certainly” take the COVID-19 vaccine, and 28.5% “probably” [8], which would be below the European Commission’s expectations in terms of vaccination coverage. In the United States, the report by Gharpure et al. showed that a median of 37.5% of the participating long-term care staff had accepted the first shot of COVID-19 vaccine (between mid-December 2020 and mid-January 2021), demonstrating a low response to the vaccination campaign by these healthcare workers [9].

Understanding vaccine hesitancy locally and its determinants is crucial to enhance the impact of vaccination strategies, as outcomes can be affected by local factors related to the given contexts, vaccines, and populations [10,11]. Identifying the determinants of vaccine hesitancy in the hesitant subgroup and then tailoring the vaccination campaign to fit this subgroup is essential [10].

The present study therefore focused on Belgian hospital staff at the beginning of the vaccination campaign, aiming to evaluate attitudes towards COVID-19 vaccination and determinants of acceptance. A secondary objective was to identify the characteristics of the hesitant subgroup (i.e., reasons for and against vaccination) and their information needs and communication preferences so as to further tailor the local vaccination campaign.

## 2. Materials and Methods

### 2.1. Study Design and Population

A cross-sectional study was conducted among hospital staff at CHU UCL Namur, a hospital center in Belgium with three sites (936 accredited beds) and around 4600 staff members. All staff members were invited to complete an online survey between 6 and 20 January 2021, including medical and non-medical staff and volunteers. People were informed about and invited to participate in the survey through different channels: email, intranet, COVID-19 internal newsletter, posters and leaflets, special note on salary slip, etc. A reminder email was sent 10 days after the survey started. The online survey was hosted on Qualtrics software, Version 01/2021 (Qualtrics, Copyright © 2021. Provo, UT, USA. Available at https://www.qualtrics.com; accessed on 5 January 2021).

The aim of the present study was to survey all the staff members and, therefore, a sample size calculation prior to conducting the study was not performed.

### 2.2. Questionnaire Development and Outcome

The questionnaire was developed based on previous surveys [12,13] conducted in the context of the 2009 H1N1 flu pandemic, which was the closest context to the COVID-19 pandemic, especially in terms of speed of vaccine development. The questionnaire was pilot tested with 5 people from the general population to assess the understandability of the questions, and with 7 healthcare professionals (including nurses, pharmacists, and specialist physicians, including infectious disease specialists) from and outside the CHU UCL Namur to assess the understandability and appropriateness of the questions in the current context of COVID-19 pandemic and vaccination.

The main outcome was the attitude towards COVID-19 vaccination based on the following question: *Are you considering getting the COVID-19 vaccine?* Participants had to answer on a five-point Likert scale (certainly not; probably not; I don’t know; yes, probably; yes, certainly). Additional questions aimed to collect data on potential determinants of attitude towards vaccination, information needs, and communication preferences.

First, the Health Belief Model (HBM) was used as the theoretical framework to assess reasons associated with the attitude towards COVID-19 vaccination. This model, developed by Hochbaum and Rosenstock, aims to “understand why and under what conditions people take action to prevent, detect and diagnose disease” [14]. It has been widely used as a theoretical framework to study vaccine hesitancy in multiple contexts, including vaccination against H1N1 flu and COVID-19 [15,16,17]. The HBM model includes five components: perceived susceptibility, perceived seriousness, perceived benefits of taking action, perceived barriers to taking action, and cues to action [14]. Supplementary Table S1 lists the questions that were used in the questionnaire with regard to the five HBM components.

Second, additional variables used in the analysis included socio-demographic data, data related to the job and hospital site, contact with non-COVID-19 and COVID-19 patients in the workplace, vaccination against seasonal flu in 2020, perceived personal health status, knowledge about COVID-19 vaccine, and personal experience with COVID-19 as well as in relation to family members, close relatives, and colleagues (Supplementary Table S2).

Regarding information needs and communication preferences, one question assessed the perceived sufficiency in terms of information on the COVID-19 vaccine and vaccine development. Finally, respondents were asked to rate their level of trust in several sources of information regarding the COVID-19 vaccines (both internal and external to the hospital; Supplementary Table S2).

### 2.3. Statistical Analysis

The exclusion criteria for data analysis were (1) a missing value for the attitude towards COVID-19 vaccination; (2) a missing value for the age group or the gender; and (3) a missing value for the work site.

Continuous variables were summarized using medians [P_25_; P_75_], and categorical variables using numbers and proportions.

To assess factors associated with attitude towards COVID-19 vaccination, from “certainly not” to “yes, certainly”, ordinal logistic regression was used. First, a univariate model was performed. All variables in univariate analysis associated with *p* < 0.15 were then included in a multivariable model. Stepwise selection based on the Akaike information criterion was then applied to select the final multivariable model. The goodness-of-fit of the final multivariable model was assessed using the Lipsitz test, the Hosmer–Lemeshow test for ordinal models, and the Pulkstenis–Robinson chi-squared test [18]. Due to correlation between a number of items, several scores were computed (Appendix A).

All analyses were performed using R software, version 4.0.2 [19]. *p* < 0.05 was considered to indicate statistical significance.

## 3. Results

### 3.1. Respondents’ Characteristics and Intention to Get the COVID-19 Vaccine

In total, 1141 respondents agreed to participate in the study and had completed the questionnaire by January 20, 2021. Nine respondents had to be excluded (missing value for work site (*n* = 8) and gender (*n* = 1)); 1132 respondents were therefore included in the analysis.

The characteristics of respondents are shown in Table 1. The majority of respondents (72.7%) were female, and the sample was diverse with regard to age, profession, and hospital site. This sample was, overall, representative of the hospital staff in relation to age, gender, and work categories, except that nurses and nursing assistants were under-represented (i.e., 28.5% of respondents were nurse or nursing assistants, while they comprised approximately 41.6% of hospital staff members). Almost 60% of respondents had previous or ongoing contact with COVID-19 patients, and 20.5% never had contact with patients. Regarding experience with COVID-19, 23.3% of the respondents had been infected with SARS-CoV-2 (mainly with no, mild, or moderate symptoms), while 86.7% knew at least one colleague who had been infected.

Fifty-eight percent and 4.9% said that they would certainly and certainly not get vaccinated, respectively; 37.1% can be categorized as hesitants, i.e., said that they would probably (22.1%) or probably not (5.9%) get vaccinated, or did not know (9.1%) (Table 1).

### 3.2. Determinants of Attitude towards COVID-19 Vaccination

Factors associated with a positive attitude towards COVID-19 vaccination in the multivariable ordinal logistic regression are shown in Table 2 and included completing the questionnaire later, being older, being a medical doctor as compared to being a member of the administrative, technical, or logistic staff, or being vaccinated against seasonal flu. Factors associated with a negative attitude towards COVID-19 vaccination included being a nurse or a nursing assistant (as compared to being a member of the administrative, technical, or logistic staff) and being female (Table 2). Among factors related to COVID-19 experience, knowing at least one colleague having had a SARS-CoV-2 infection, and even more so in the case of hospitalization, was associated with a positive attitude, while previous personal infection was not.

Among HBM-related factors, perceived benefits (i.e., personal benefits, benefits for others and collective benefits) and cues to action items (i.e., perception of being sufficiently informed) were associated with positive attitudes towards COVID-19 vaccination. Perceived susceptibility and seriousness were partially associated with positive attitudes: having no opinion on one’s probability of being infected or re-infected, and having no opinion on the consequences for a relative being infected or re-infected were associated with negative attitudes toward vaccination (Table 2).

### 3.3. Characteristics of the Hesitant Subgroup: Reasons for and against COVID-19 Vaccination 

**Figure 1:**
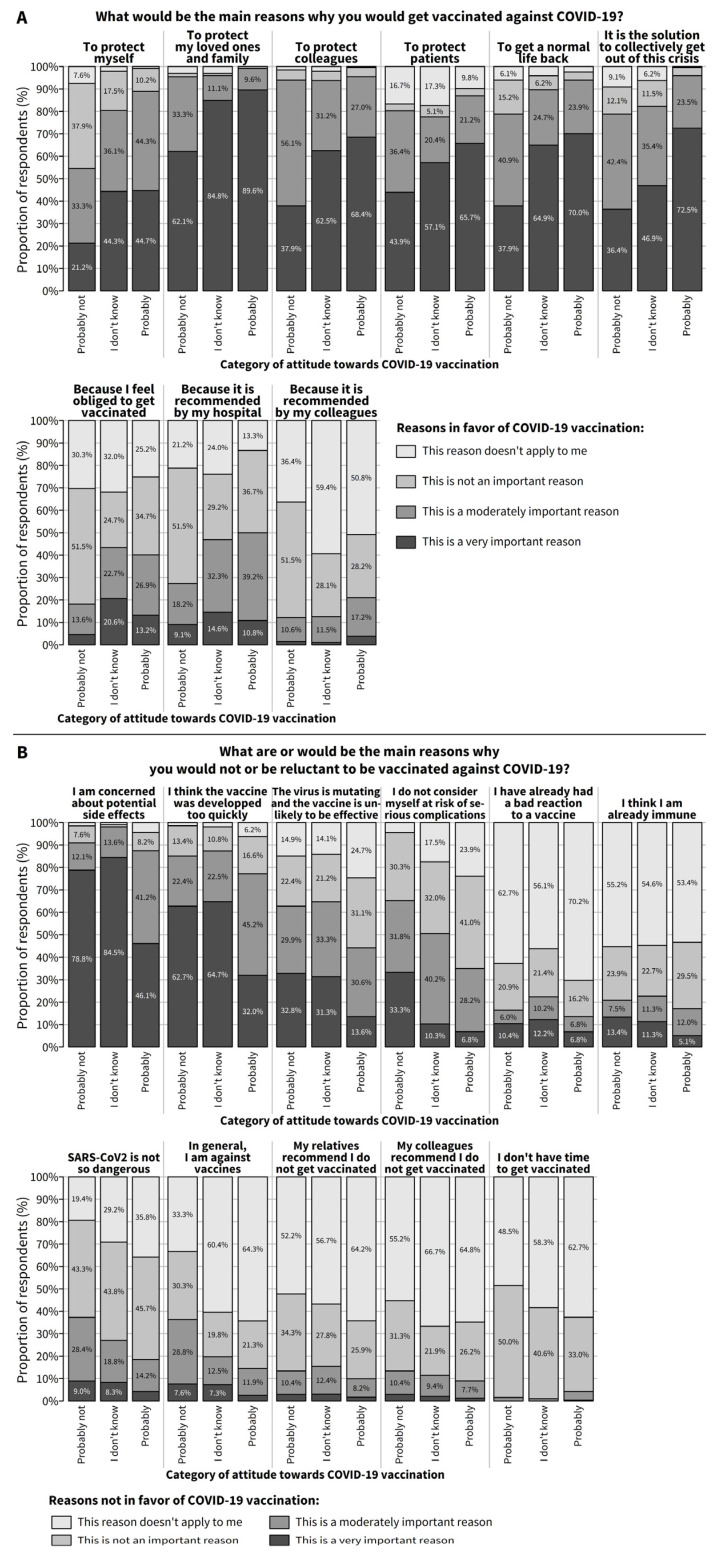
Among respondents who were hesitant about COVID-19 vaccination, distribution of the importance of each reason why a respondent (**A**) would get the COVID-19 vaccine (*N* = 420) or (**B**) would not get the COVID-19 vaccine (*N* = 418).

Figure 1 shows, in the hesitant subgroup (*N* = 420), the importance of several reasons why a respondent would (A) or would not (B) get the COVID-19 vaccine. The most important reasons why a hesitant respondent would get vaccinated (and the overall percentage for whom this was rated as a very important reason) included the protection of loved ones and family (84.1%), of colleagues (62.1%) or of patients (60.1%), to get back to normal life (63.7%), to collectively get out of the crisis (60.6%) and, to a lesser extent, to protect themselves (40.8%). Feeling obliged to get vaccinated and vaccination being recommended by the hospital or by colleagues were not considered important reasons.

As for reasons why hesitant respondents would not get vaccinated, the main reasons (and the percentage for whom this was rated as a very important reason) were concerns about potential side effects (60.9%) and the impression that the vaccine was developed too quickly (45.1%). To a lesser extent, other reasons included the impression that the vaccine may not be effective against mutants (very or moderately important reason for 52.4% of respondents), and the fact that they did not consider themselves at risk of serious complications from COVID-19 (very or moderately important reason for 43.8% of respondents). Other reasons were considered either not important or not applicable for the majority of the respondents.

### 3.4. Need for Information about COVID-19 Vaccine and Trust in Different Sources of Information among Hesitant Respondents

**Figure 2:**
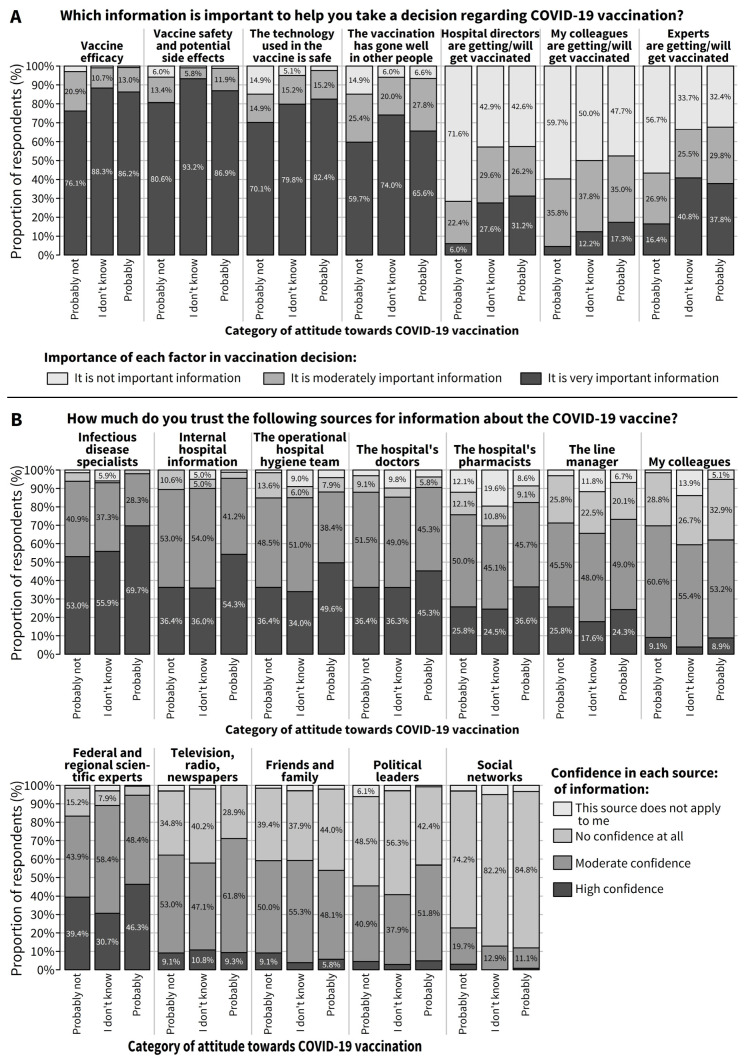
Among respondents who were hesitant about COVID-19 vaccination, (**A**) importance of different types of information to help make a decision regarding COVID-19 vaccination (*N* = 417) and (**B**) level of trust in different sources of information about COVID-19 vaccines (*N* = 416).

On the one hand, among the 417 hesitant respondents who answered the question on their need for information about the COVID-19 vaccine, most consider that information on vaccine efficacy, vaccine safety and potential side effects, the safety of the technology used in the vaccine, and knowing that the vaccination went well in other people is very important information to make a decision regarding COVID-19 vaccination (85.1%, 87.4%, 79.8%, and 66.7%, respectively) (Figure 2A). Conversely, knowing that experts, hospital directors, or colleagues are or will be vaccinated is not important information for 36.7%, 47.5%, and 50.2% of the hesitant respondents, respectively.

On the other hand, among the 416 hesitant respondents who answered the question on their trust in different sources of information to provide information about the COVID-19 vaccine, a high degree of confidence is observed in the majority of information sources from the hospital and in a minority of sources outside the hospital. The information sources from the hospital include infectious disease specialists (high confidence for 63.6% and moderate confidence for 32.5%), internal information from the hospital (high confidence for 47.0% and moderate confidence for 46.2%), the operational hospital hygiene team (high confidence for 43.6% and moderate confidence for 43.1%), the hospital doctors (high confidence for 41.6% and moderate confidence for 47.2%), the hospital pharmacists (high confidence for 31.9% and moderate confidence for 46.2%) and, to a lesser extent, the line manager or colleagues (Figure 2B). Regarding sources of information on the COVID-19 vaccine outside the hospital, a non-negligible proportion of hesitant respondents have a high degree of confidence in federal and regional scientific experts (high confidence for 41.4% and moderate confidence for 50.1%), but only a small proportion have a high degree of confidence in information coming from television, radio, or newspapers (no confidence at all for 32.6%), friends and family (no confidence at all for 41.7%), or political leaders (no confidence at all for 46.9%), and almost none have a high degree of confidence in information coming from social media (no confidence at all for 82.5%).

## 4. Discussion

### 4.1. Summary of Results

In our sample of 1132 hospital staff, 80.1% of respondents reported that they would certainly or probably get vaccinated against COVID-19. We identified several factors associated with a positive attitude towards COVID-19 vaccination, including being older, being a physician, previous influenza vaccination, and having a colleague previously infected with SARS-CoV-2. Several HBM-related items (relating to perceived susceptibility and seriousness of the disease, perceived benefits of taking action, and cues to action) were also associated with a positive attitude towards vaccination. The hesitant subgroup represented 37.1% of the respondents. Our results provide detailed information on the main reasons that matter to them for final vaccine acceptance or refusal, and the information they need and from whom in order to make their decision. What mattered most to respondents was information on vaccine efficacy, vaccine safety, and previous vaccination of others.

### 4.2. Vaccination Intention and Factors Associated with Positive Attitude (or Hesitancy)

A few other studies worldwide have evaluated the COVID-19 vaccination intention of HCPs. In the USA, a survey assessed the attitudes of all clinical and non-clinical staff at a children’s hospital [20]. Other studies, not specific to hospitals, examined HCPs’ attitudes either in Greece [21] or in France and the French-speaking part of Belgium and Canada [8]. Overall, these studies reported results similar to ours, with vaccination acceptance rates ranging from 78.5% to 80.1%. Several factors reported to be associated with vaccine acceptance or hesitancy were similar to our findings. Older age was associated with higher rates of vaccine acceptance in several studies [20,21]. Females were reported to be more reluctant about vaccination in both HCP [20] and general population studies [22,23]. The association between history of influenza vaccination and COVID-19 vaccine acceptance [8] and variations between occupations [21,24] have also been reported elsewhere, as has a rise in COVID-19 vaccine acceptance over time [25]. The fact that nurses and nursing assistants had negative attitudes towards COVID-19 vaccination (compared to members of the administrative, technical, or logistic staff) is a matter of concern and suggests that vaccination communication efforts should also target this subgroup. Finally, similar to the study among USA hospital staff, we found an association between vaccination acceptance and the HBM factors “perceived susceptibility” and “perceived seriousness”, although in our study, seriousness for a relative (and not for ourselves) was significant [20]. In our study, we also reported an influence of the factor “cues to action”, specifically the importance of perceived sufficiency of information. This suggests that the communication strategy could now be also directed at those “who do not yet feel sufficiently informed”.

Attitudes towards COVID-19 vaccination in the general population have been evaluated more extensively, and it is interesting to address similarities and differences in factors associated with vaccine reluctance in the general population as compared to hospital staff. Overall, among the general population, vaccine acceptance was associated with male gender, educational level, history of chronic disease and white, non-Hispanic people [22,23]. Interestingly, personal history of COVID-19 infection was not associated with vaccine acceptance [22], which is similar to what we observed in our sample. In our study, we showed that some factors of the Health Belief Model were associated with vaccination intention. HBM factors were also investigated in a survey on COVID-19 vaccine acceptance conducted among the Hong Kong population. Perceived severity, perceived benefits of the vaccine, cues to action, self-reported health outcomes, and trust in the healthcare system or vaccine manufacturers were positively associated with vaccine acceptance, while perceived access barriers and harm were negatively associated with vaccine acceptance [16]. This is in line with a recent factorial survey experiment, where all HBM components were found to influence vaccination intention. The HBM components in their model explained 59% of the variance in intentions [17]. Together with our results, this shows that this model is helpful in examining factors associated with vaccine hesitancy.

### 4.3. Characteristics of the Hesitant Subgroup and Key Messages for Further Tailoring the Vaccination Campaign

Our study also evaluated information needed and reasons for or against vaccination among hesitant people. The information ranked as being most important by our sample was data on vaccine efficacy, potential side effects, and safety of vaccine technology. This is in line with a study among 2133 Egyptian medical students who ranked deficient data regarding the vaccine’s side effects and insufficient information on the vaccine as the two main barriers to COVID-19 vaccination [26]. Concerns about vaccine safety were also an important predictor of vaccine acceptance in two studies evaluating HCPs’ attitudes [8,21]. Concerns about vaccine safety are not specific to COVID-19 vaccines and were reported for other vaccines among both HCPs and the general population [27,28]. These data confirm that providing information on the risk–benefit ratio of vaccination is of paramount importance [29]. These concerns must be addressed as soon as possible because the longer people continue to be hesitant, the fewer will get vaccinated [29,30]. Another important factor for hesitant respondents was knowing that vaccination of others has gone well. This is in line with both National Institutes of Health and Centers for Disease Control and Prevention guidelines on COVID-19 vaccine communication, which recommend sharing the positive experience of vaccinated people to enhance popular trust in the vaccine [29,31].

In terms of the type of communication, a communicating strategy that is people centered and uses first person narratives with emotional language is recommended [32]. Adapting the communication to each population is also important. With this in mind, guidelines recommend the use of “trusted messengers” to spread information [29]. In our sample, hesitant respondents relied more on information coming from within the hospital than on information from outside the hospital. Our data clearly suggest that these “trusted messengers” in our context are infectious disease specialists, the operational hospital hygiene team, and hospital doctors. It is interesting to note that this result is highly dependent on the country culture. Indeed, in a study conducted in Hong Kong, the government’s recommendations were the strongest predictor of vaccination [16].

### 4.4. Strengths and Limitations

First, the present survey was conducted within a unique hospital network—yet with multiple sites—and the data may therefore not be representative of the attitudes of hospital staff across Belgium as a whole. Our intention was to understand vaccine hesitancy locally so as to tailor the local vaccination campaign, and this approach is strongly recommended by expert groups on vaccine hesitancy [10].

Second, it proved difficult to set up a tailored and timely local vaccination campaign in January and February, as initially planned, because regional health authorities urgently requested to know, by 15 January, the exact number of hospital staff willing to be vaccinated through the hospital vaccination hub. We have so far achieved vaccination of 75% of hospital staff, but it is likely that a higher percentage will be reached in the coming weeks through access for our staff to other vaccination rounds.

Third, even though we reached a relatively high number of respondents in a short period of time, response rate was 25%. Our sample was overall representative of the hospital staff in relation to age, gender, and work categories, except that nurses and mainly nursing assistants were under-represented. The low participation rate among the latter might be partly explained by the fact that most do not have a professional e-mail address and do not use computers for their daily work. Consequently, we had to pool them with nurses, but attitudes might be different between these two groups. Overall COVID-19 vaccine acceptance may therefore have been overestimated. This is likely a limited issue, as the 75% vaccination rate achieved during the first vaccination round (in January and February) is close to the intentions observed in our survey (i.e., 80.1% of respondents answering that they would certainly or probably get the COVID-19 vaccine). The exact percentage might be higher as some members of staff had the opportunity to get vaccinated outside the hospital. Despite this limitation, we were able to evaluate the association between many different factors and attitude towards vaccination—several of these factors being scarcely considered in other studies.

Fourth, only the mRNA vaccines were available when the data were collected. Consequently, we did not assess the impact of the vaccine type on vaccination intention. Vaccination intention and associated factors may differ between vaccines.

Finally, this survey was launched only 2 weeks after the approval of the first vaccine against COVID-19 by the European Medicines Agency and before the start of vaccination at the hospital. The measure of the attitude towards vaccination in the present study was performed at a certain time point in this context, and we cannot exclude that respondents’ opinion will not vary over time with the arrival of new information about the vaccines, the vaccination, or increasing knowledge on COVID-19.

## 5. Conclusions

Hospital staff are a priority target group in the COVID-19 vaccination strategy. By conducting a survey among hospital staff at a three-site hospital center, we were able to better understand vaccine hesitancy locally and its determinants. Among hesitant staff, concerns about potential side effects and the impression that the vaccine was developed too quickly were the main reasons against COVID-19 vaccination. The key factors in the decision process were data on vaccine efficacy and safety, and knowing that vaccination went well in others. These data are helpful to further tailor the communication campaign and ensure sufficient vaccination coverage among hospital staff. At our hospital, this will mean focusing on staff members who are younger than 45, female, and nurses or nurse assistants. A more qualitative research approach would be helpful to complement the identification of enablers and barriers for nursing assistants.

## Figures and Tables

**Table 1:** Description of respondents’ characteristics (*N* = 1132).

Variables	Total (*N* = 1132) *n* (%)	Attitude towards COVID-19 Vaccination		
		Will Certainly Not Get the Vaccine (*n* = 55) *n* (%)	Is Hesitant to Get the Vaccine (*n* = 420) *n* (%)	Will Certainly Get the Vaccine (*n* = 657) *n* (%)
**Period of questionnaire completion**				
1 (6 January to 11 January 2021)	548 (48.4)	31 (56.4)	221 (52.6)	296 (45.1)
2 (12 January to 15 January 2021)	247 (21.8)	12 (21.8)	86 (20.5)	149 (22.7)
3 (16 January to 20 January 2021)	337 (29.8)	12 (21.8)	113 (26.9)	212 (32.3)
**Socio-demographic variables**				
Age group				
<35 years	321 (28.4)	24 (43.6)	154 (36.7)	143 (21.8)
35–44 years	275 (24.3)	22 (40.0)	121 (28.8)	132 (20.1)
45–54 years	274 (24.2)	5 (9.1)	88 (21.0)	181 (27.5)
≥55 years	262 (23.1)	4 (7.3)	57 (13.6)	201 (30.6)
Female	823 (72.7)	43 (78.2)	341 (81.2)	439 (66.8)
Work ^‡^				
Medical doctor	185 (16.5)	0 (0.0)	32 (7.6)	153 (23.6)
Nurse or Nursing assistant	319 (28.5)	17 (30.9)	128 (30.5)	174 (26.9)
Paramedical staff	206 (18.4)	5 (9.1)	89 (21.2)	112 (17.3)
Administrative, technical, or logistic staff	329 (29.3)	29 (52.7)	149 (35.6)	151 (23.3)
Other	82 (7.3)	4 (7.3)	21 (5.0)	57 (8.8)
Hospital site				
Teaching hospital (one site)	649 (57.3)	25 (45.5)	217 (51.7)	407 (61.9)
Non-teaching hospital (two sites)	468 (41.3)	29 (52.7)	197 (46.9)	242 (36.8)
Working at several sites	15 (1.3)	1 (1.8)	6 (1.4)	8 (1.2)
**COVID-19 experience**				
Contact with non-COVID-19 and COVID-19 patients *				
Not in contact with patients at all	232 (20.5)	9 (16.7)	106 (25.3)	117 (17.8)
Contact with non-COVID-19 patients	230 (20.4)	16 (29.6)	87 (20.8)	127 (19.4)
Previously in contact with COVID-19 patient, but not now	333 (29.5)	16 (29.6)	114 (27.2)	203 (30.9)
Current contact with COVID-19 patients	334 (29.6)	13 (24.1)	112 (26.7)	209 (31.9)
Personal infection with SARS-CoV-2 and COVID-19 symptoms **				
Never infected	866 (76.8)	43 (79.6)	312 (74.5)	511 (78.0)
Yes, with no, mild, or moderate symptoms	214 (19.0)	10 (18.5)	82 (19.6)	122 (18.6)
Yes, with major symptoms or hospitalization	48 (4.3)	1 (1.9)	25 (6.0)	22 (3.4)
Infection of people living under the same roof with SARS-CoV-2 and COVID-19 symptoms ***				
No-one infected	847 (75.2)	43 (78.2)	301 (72.2)	503 (76.8)
Yes, with no, mild or moderate symptoms	268 (23.8)	11 (20.0)	112 (26.9)	145 (22.1)
Yes, and required hospitalization	12 (1.1)	1 (1.8)	4 (1.0)	7 (1.1)
Infection of colleagues with SARS-CoV-2 and COVID-19 symptoms ^†^				
No-one infected	149 (13.3)	13 (23.6)	66 (15.9)	70 (10.7)
Yes, with no, mild, or moderate symptoms	892 (79.5)	41 (74.5)	324 (78.3)	527 (80.7)
Yes, and required hospitalization	81 (7.2)	1 (1.8)	24 (5.8)	56 (8.6)
**Attitude towards COVID-19 vaccination**				
Certainly not getting the COVID-19 vaccine	55 (4.9)			
Probably not getting the COVID-19 vaccine	67 (5.9)			
I don’t know	103 (9.1)			
Yes, probably getting the COVID-19 vaccine	250 (22.1)			
Yes, certainly getting the COVID-19 vaccine	657 (58.0)			

* 3 missing values (0.3%); ** 4 missing values (0.4%); *** 5 missing values (0.4%); ^†^ 10 missing values (0.9%); ^‡^ 11 missing values (1.0%).

**Table 2:** Factors associated with positive attitudes towards COVID-19 vaccination in univariate and multivariable ordinal logistic regression.

Variables	*n* (%) or Median [P_25_; P_75_]	Univariate Analysis (*N* = 1132)		Multivariable Analysis (*N* = 1077)	
		OR [95% CI]	*p*-Value	OR [95% CI]	*p*-Value
**Period of questionnaire completion**					
1 (Jan 6 to Jan 11, 2021)	548 (48.4)	1.00		1.00	
2 (Jan 12 to Jan 15, 2021)	247 (21.8)	1.29 [0.97; 1.74]	0.084	1.41 [0.98; 2.02]	0.063
3 (Jan 16 to Jan 20, 2021)	337 (29.8)	1.39 [1.07; 1.82]	0.016	2.11 [1.52; 2.95]	<0.001
**Socio-demographic variables**					
Age group					
<35 years	321 (28.4)	1.00		1.00	
35–44 years	275 (24.3)	1.19 [0.88; 1.60]	0.257	1.18 [0.83; 1.69]	0.348
45–54 years	274 (24.2)	2.57 [1.87; 3.54]	<0.001	2.30 [1.56; 3.39]	<0.001
≥55 years	262 (23.1)	4.22 [2.98; 6.02]	<0.001	3.45 [2.20; 5.47]	<0.001
Female		0.47 [0.36; 0.62]	<0.001	0.48 [0.34; 0.68]	<0.001
Work ^†††^					
Medical doctor	185 (16.5)	5.77 [3.80; 8.98]	<0.001	1.88 [1.07; 3.36]	0.030
Nurse or Nursing assistant	319 (28.5)	1.35 [1.01; 1.81]	0.042	0.68 [0.47; 0.98]	0.040
Paramedical staff	206 (18.4)	1.41 [1.02; 1.96]	0.040	1.03 [0.70; 1.53]	0.883
Administrative, technical, or logistic staff	329 (29.3)	1.00		1.00	
Other	82 (7.3)	2.54 [1.54; 4.27]	<0.001	1.08 [0.56; 2.12]	0.830
Working in a teaching hospital		1.61 [1.28; 2.02]	<0.001		
**Personal health status**					
Presence of COVID-19 risk factor(s) ^†^	256 (22.7)	1.31 [1.00; 1.73]	0.053		
Seasonal flu vaccination in 2020 ^†^	564 (50.0)	5.20 [4.05; 6.69]	<0.001	3.02 [2.22; 4.12]	<0.001
**COVID-19 experience**					
Contact with non-COVID-19 and COVID-19 patients ***					
Not in contact with patients at all	232 (20.5)	1.00			
Contact with non-COVID-19 patients	230 (20.4)	1.18 [0.84; 1.66]	0.349		
Previously in contact with COVID-19 patients, but not now	333 (29.5)	1.37 [0.99; 1.88]	0.056		
Current contact with COVID-19 patients	334 (29.6)	1.48 [1.08; 2.05]	0.016		
Personal infection with SARS-CoV-2 and COVID-19 symptoms ^†^					
Never infected	866 (76.8)	1.00			
Yes, with no, mild, or moderate symptoms	214 (19)	0.94 [0.71; 1.26]	0.678		
Yes, with major symptoms or hospitalization	48 (4.3)	0.77 [0.46; 1.31]	0.330		
Infection of colleagues with SARS-CoV-2 and COVID-19 symptoms ^††^					
No-one infected	149 (13.3)	1.00		1.00	
No, mild, or moderate symptoms	892 (79.5)	1.72 [1.24; 2.39]	0.001	1.69 [1.11; 2.55]	0.014
Required hospitalization	81 (7.2)	2.61 [1.52; 4.59]	0.001	2.69 [1.37; 5.41]	0.005
**HBM—perceived susceptibility**					
Probability of being infected/re-infected *					
Fairly or very likely	439 (38.8)	1.00		1.00	
Not really likely	243 (21.5)	0.63 [0.46; 0.86]	0.004	0.78 [0.54; 1.15]	0.210
Not at all likely	15 (1.3)	0.12 [0.04; 0.34]	<0.001	0.32 [0.09; 1.19]	0.087
No opinion	434 (38.4)	0.53 [0.40; 0.68]	<0.001	0.69 [0.50; 0.95]	0.025
**HBM—perceived seriousness**					
Expected consequences in case of future infection *					
Quite or very serious	180 (15.9)	1.00			
Not really serious	434 (38.4)	0.44 [0.30; 0.63]	<0.001		
Not at all serious	50 (4.4)	0.21 [0.11; 0.40]	<0.001		
No opinion	467 (41.3)	0.46 [0.32; 0.67]	<0.001		
Expected consequences in case of future infection for a relative **					
Very serious	223 (19.7)	1.00		1.00	
Quite serious	529 (46.8)	0.57 [0.41; 0.79]	0.001	0.68 [0.46; 1.01]	0.060
No consequences for any relatives	139 (12.3)	0.40 [0.26; 0.61]	<0.001	0.37 [0.22; 0.62]	<0.001
No opinion	239 (21.2)	0.36 [0.25; 0.52]	<0.001	0.45 [0.29; 0.71]	0.001
**HBM—perceived benefits of taking action**					
Score for perceived personal benefits: for personal protection, to be able to get back to a more normal life (−2 to 2) ^††^	1.0 [0.0; 2.0]	2.37 [2.09; 2.69]	<0.001	1.35 [1.15; 1.58]	<0.001
Score for perceived benefits for others: to protect relatives, to protect patients, to protect colleagues (−3 to 3) ^††^	3.0 [2.0; 3.0]	1.95 [1.76; 2.16]	<0.001	1.38 [1.22; 1.57]	<0.001
It is a solution to collectively get out of this crisis ^††^					
Not important or this reason does not apply to me	83 (7.4)	0.12 [0.07; 0.20]	<0.001	0.23 [0.13; 0.39]	<0.001
Moderately important	188 (16.8)	1.00		1.00	
Very important	851 (75.8)	6.06 [4.45; 8.28]	<0.001	3.21 [2.25; 4.57]	<0.001
**HBM—Cues to action**					
Score for perception of being sufficiently informed about vaccine efficacy, side effects and safety, and development process (−3 to 3) ^‡^	−1 [−3; 1]	1.46 [1.38; 1.55]	<0.001	1.22 [1.13; 1.32]	<0.001
**Knowledge about COVID-19 vaccine**					
Score for knowledge about COVID-19 vaccine (0 to 4) ^‡‡^	1.0 [1.0; 2.0]	1.74 [1.55; 1.96]	<0.001	1.12 [0.96; 1.30]	0.139

* 1 missing value (0.1%); ** 2 missing values (0.2%); *** 3 missing values (0.3%); ^†^ 4 missing values (0.4%); ^††^ 10 missing values (0.9%); ^†††^ 11 missing values (1.0%); ^‡^ 13 missing values (1.1%); ^‡‡^ 16 missing values (1.4%); OR: odds ratio; 95% CI: 95% confidence interval; COVID risk factors included obesity (body mass index ≥30 kg/m^2^, diabetes, hypertension, chronic cardiovascular, pulmonary, renal or hepatic diseases, cancer diagnosed <5 years ago); Multivariable model goodness-of-fit tests: Lipsitz test (*p* = 0.608), Hosmer–Lemeshow test (*p* = 0.409), Pulkstenis–Robinson chi-squared test (*p* = 0.275).

## Data Availability

The data sets used and/or analyzed during the present study are available on request from Séverine Henrard. The data are not publicly available due to them containing information that could compromise research participant privacy.

## Supplementary Materials

The following are available online at https://www.mdpi.com/article/10.3390/vaccines9050469/s1, Table S1: Questions related to the five Health Belief Model components in the questionnaire, Table S2: Additional data collected.

## Appendix A. Scores Computed for the Analysis of Factors Associated with Positive Attitudes towards COVID-19 Vaccination

Due to correlation between a number of items, several scores were computed.

First, a score for perceived personal benefits of COVID-19 vaccination was computed based on the questions relating to the reasons why a respondent would get vaccinated against COVID-19. The perceived personal benefits score was constructed based on two questions (to protect myself, and to be able to get back to a more normal life). One point per question was given if the respondent responded “this reason is very important”, while one negative point was given for each question if the answer was “this reason is not important”. This resulted in a score ranging from −2 to 2.

Second, the perceived benefits for others score was constructed in a similar way, based on the following three reasons: to protect my loved ones and family, my patients, and my colleagues. One point per question was given if the respondent responded “this reason is very important”, while one negative point was given for each question if the answer was “this reason is not important”. This resulted in a score ranging from −3 to 3.

The third score related to knowledge about COVID-19 vaccine. Four true or false questions were asked about the COVID-19 vaccine and vaccine development. A score counting the correct answers was computed, ranging from 0 to 4.

A score for perception of being sufficiently informed about vaccine efficacy, side effects and safety, and development was computed. The answer “I am sufficiently informed” to each question counted for one point, while the answer “I am not sufficiently informed” counted for one negative point. This resulted in a score ranging from −3 to 3.
