# Supplementary material for: Attitudes towards COVID-19 Vaccination among Hospital Staff—Understanding What Matters to Hesitant People
Source: Vaccines (Basel). 2021 May 6;9(5):469. doi: 10.3390/vaccines9050469 (PMC8148217; doi:10.3390/vaccines9050469)
Supplement: Supplementary file 1 [file vaccines-09-00469-s001.zip › vaccines-1192974-supplementary.pdf]

Table S1. Questions related to the five Health Belief Model components in the questionnaire.

Health Belief Model (HBM) Component	Related Questions in the Questionnaire
**Perceived susceptibility**	How likely do you think you are to become infected or re-infected with SARS-CoV-2? *(Possible answers: not at all likely; not really likely; no opinion; fairly likely; very likely)*
**Perceived seriousness**	1) If you were to become infected or re-infected with SARS-CoV-2, what health consequences do you think this could have? *(Possible answers: not at all serious; not really serious; no opinion; quite serious; very serious)*<br>2) If any of your close relatives were to become infected or re-infected with SARS-CoV-2, do you think that for at least one of them, the consequences for their health could be serious? *(Possible answers: yes, very serious consequences; yes, quite serious consequences; no, for any of my relatives; no opinion)*
**Perceived benefits of taking action**	What are or would be the reasons why you would get vaccinated against COVID-19? Indicate how important each of the following reasons is to you, regardless of your intention to get vaccinated. *(Possible answers: very important reason; moderately important reason; not important reason; does not apply to me)*<br>• To protect myself personally;<br>• To protect patients/residents;<br>• To protect my loved ones, my family;<br>• To protect my colleagues;<br>• Because it is the solution to collectively get out of this epidemic (it is a solution to society's problem);<br>• To be able to get back to a more normal life (no more lockdown, no restriction of social contact, freedom to travel, to go out, etc.);<br>• Other, please specify:
**Perceived barriers to taking action**	What are or would be the reasons why you would not or would be reluctant to get vaccinated against COVID-19? Indicate how important each of the following reasons is to you, regardless of your intention to get vaccinated. *(Possible answers: very important reason; moderately important reason; not important reason; this reason does not apply to me)*<br>• I think the vaccine was developed too quickly<br>• I am concerned about potential side effects<br>• I have had a bad reaction to a vaccine in the past<br>• I don't have time to get vaccinated<br>• In general, I am against vaccines<br>• The virus is mutating and I think the vaccine is probably not going to be effective<br>• Other, please specify:
**Cues to action**	1) Please indicate whether you are sufficiently informed about the following topics:<br>• Vaccine efficacy<br>• Safety and potential side effects of the vaccine<br>• The vaccine development process<br>2) What factors helped you or would help you make the decision to get the COVID-19 vaccine? Select the level of importance for each of the following statements below *(Possible answers: very important; moderately important; not important)*<br>• Information on the safety and potential side effects of the vaccine<br>• Information on vaccine efficacy<br>• To know whether the vaccination went well in other people<br>• Knowing that my colleagues are or will be vaccinated<br>• Knowing that experts are or will be vaccinated<br>• Knowing that members of management are or will be vaccinated<br>• Knowing that the technology used in the vaccine is safe<br>• Other, please specify:

Table S2. Additional data collected.

**Socio-Demographic Data**
Age:
Gender:
Size of the household (number of household members aged <12 years, aged 12-17 years, aged 18-39 years, aged 40-64 years, aged ≥65 years)
Information about work situation
Main work site
Occupation (physician, nurse, nursing assistant, paramedic, administrative, technical or logistic staff, other)
Administrative status I (employee, self-employed, assistant, trainee, agency staff, volunteer, other)
Administrative status II (type of employment contract; non-medical manager, management members, other)
Contact with patients
Present or past contact with COVID-19 patients (if yes, in which department)
Vaccination against seasonal influenza in 2020
Perceived health status of oneself, friends and family
Existence of chronic health conditions (obesity (Body Mass Index >30); hypertension; cardiovascular disease, pulmonary disease; hepatic disease; cancer (diagnosed less than five years ago))
Overall health status
Living with or being close to someone particularly vulnerable to SARS-CoV-2 (older adults, people living with the chronic conditions listed above)
Knowledge about COVID-19 (two statements to rate as “correct”, “do not know” or “not correct”)
2. In the population, COVID-19 causes a respiratory tract infection that, in most cases, resolves within a few days or even weeks. In some cases, prolonged symptoms may occur.
3. In severe cases, in addition to respiratory problems, the infection can lead to cardiac, neurological, and clotting disorders.
Personal experience with COVID-19
Have you ever been considered as infected with SARS-CoV-2?
If yes:
Was it confirmed by a test?
What were the symptoms?
When was the last infection?
Do you think that you are immunized against SARS-CoV-2?
Experience of family members, close relatives and colleagues with COVID-19 (the same questions were asked for each group)
Has (a family member/a close relative/a colleague) been considered as infected with SARS-CoV-2?
If yes: What were the symptoms?
Knowledge about COVID-19 vaccine (four statements to rate as “correct”, “do not know” or “not correct”
1. The European Medicines Agency's “fast-track” procedure for approving COVID-19 vaccines means that studies may include a smaller number of patients than in a normal procedure.
2. Each vaccination (person vaccinated, batch of vaccine) will be recorded in a database that will allow the information to be kept for traceability purposes but also for efficacy and safety research.
3. Available data indicate that COVID-19 vaccine provides better protection than seasonal influenza vaccines.
4. Data from clinical studies show that it will be necessary to be revaccinated every year against COVID-19.
Perceived sufficiency of information about COVID-19 vaccine
1. Please indicate whether you are sufficiently informed about the following topics:
• Vaccine efficacy
• Safety and potential side effects of the vaccine
• The vaccine development process
Level of trust in the following sources to provide information about COVID-19 vaccine:
Television, radio, newspapers
Social media
Friends and family
Political leaders
Scientific experts from the federal and regional governments
Internal information from the hospital
Infectious disease specialists
The Operational Hospital Hygiene Team
Doctors from the hospital
Pharmacists from the hospital
Your line manager
Your colleagues
